# Palmitic Acid Inhibits the Growth and Metastasis of Gastric Cancer by Blocking the STAT3 Signaling Pathway

**DOI:** 10.3390/cancers15020388

**Published:** 2023-01-06

**Authors:** Xiaojuan Yu, Wen Peng, Yaoxing Wang, Wenjun Xu, Wentong Chen, Lei Huang, Hu Xu, Xinyu He, Sheng Wang, Qianqian Sun, Wenjie Lu, Youzhi Xu

**Affiliations:** 1 College of Basic Medicine, Anhui Medical University, 81 MeiShan Road, Hefei 230032, China; 2 Department of Oncology, The People’s Hospital of Guizhou Province, #83 Zhong Shan East Road, Guiyang 550004, China; 3 Center for Scientific Research, Anhui Medical University, 81 MeiShan Road, Hefei 230032, China

**Keywords:** gastric cancer, lipidomics, palmitic acid, proliferation, p-STAT3

## Abstract

**Simple Summary:**

In this study, we investigated the effects of palmitic acid (PA) on multiple human gastric cancer cell lines and combined them with the results from clinical gastric cancer and paracancerous tissue samples. Our findings demonstrated that PA exerted anti-gastric cancer effects by regulating key molecules in the signal transduction and activation of a transcription 3 (STAT3) protein inhibitor of the activated STAT 3 (PIAS3) signaling pathway. Our results serve as a foundation for further research on the correlation between the anti-gastric cancer activity of PA and the STAT3-PIAS3 signaling pathway. Our results also represent a critical step toward understanding gastric cancer prevention and prognosis and promoting PA supplementation as a gastric cancer treatment.

**Abstract:**

Lipidomic analyses have suggested that palmitic acid (PA) is linked to gastric cancer. However, its effects and action mechanisms remain unclear. Therefore, we evaluated the effects of PA on cell proliferation, invasion, and apoptosis in human gastric cancer, as well as the role of p-STAT3 in mediating its effects. The results of the MTT and colony formation assays revealed that PA blocked gastric cancer cell proliferation in a concentration-dependent manner. The EdU-DNA assay indicated that 50 μM of PA could block gastric cell proliferation by 30.6–80.0%. The Transwell assay also confirmed the concentration dependence of PA-induced inhibitory effect on cell invasion. The flow cytometry analysis indicated that PA treatment for 18 h could induce gastric cancer cell apoptosis. The immunohistochemical staining revealed that p-STAT3 levels were higher in the gastric cancer tissues than in the control tissues. We demonstrated that PA treatment for 12 h decreased the expressions of p-STAT3, p-JAK2, N-cadherin, and vimentin, and inhibited the nuclear expression of p-STAT3 in gastric cancer cells. Finally, PA treatment (50 mg/kg) decreased gastric cancer growth (54.3%) in the xenograft models. Collectively, these findings demonstrate that PA inhibits cell proliferation and invasion and induces human gastric cancer cell apoptosis.

## 1. Introduction

Gastric cancer, a common form of cancer that includes gastric adenocarcinomas, has the fifth and third highest morbidity and mortality rates, respectively, thereby posing an enormous global health burden [1]. It is also highly prevalent in China [2]. Its frequent recurrence and metastasis have led to a poor five-year survival rate of <30% [3]. Changes in cellular behavior or epigenetic modifications may promote disease progression [4]. Most gastric cancers are initially diagnosed at an advanced stage; therefore, current treatment strategies exhibit poor clinical efficacy. Critical staging is often missed despite the discovery of several predictive and therapeutic biomarkers [5,6].

A critical feature of cancer cells is the reprogramming of metabolic pathways [7]. Lipids are hydrophobic biomolecules, and fatty acids are composed of hydrocarbon chains of different lengths [8]. Palmitic acid (PA), a 16-carbon backbone long-chain saturated fatty acid, can be obtained from our diet or endogenously synthesized; it accounts for 20% of the total fatty acids [9,10]. Concordantly, PA serves as an energy source and a signaling molecule involved in regulating various diseases [11,12]. However, it has conflicting roles in tumorigenesis and tumor development [13]. For example, PA can promote the metastasis of oral cancer [14]; however, it can also reduce the malignant proliferation of liver cancer in mouse xenografts, thereby reducing the invasiveness of cancer cells [15]. Therefore, its action mechanism in these processes requires further exploration.

Imbalanced signal transduction and transcriptional regulation play a vital role in tumorigenesis [16]. STAT3 is a pro-inflammatory and carcinogenic transcription factor linked to tumor occurrence, inflammation, and immunosuppression [17]. Abnormal STAT3 activation is implicated in tumor malignancy [18]. Receptor-associated kinases (such as JAK2 and MET) can catalyze STAT3 phosphorylation to form homo- or heterodimers, which act as transcription factors upon their nuclear translocation [19]. A member of the protein inhibitor of the activated STAT3 (PIAS3) family, PIAS3, can inhibit activated STAT3 [20,21]. PIAS3 dysregulation is crucial in inflammatory diseases and cancers, including gastric cancer [22]. However, the mechanisms by which the p-STAT3/PIAS3 pathway regulates the occurrence and development of gastric cancer remain unclear. Functional studies on critical molecules identified using a lipidomic approach may provide novel targets for the prevention and treatment of tumors [23,24] Concordantly, we reported lower PA levels in clinical gastric cancer samples than in paracancerous tissues by performing lipidomic analysis using an ultraperformance liquid chromatography–tandem mass spectrometry (UPLC–MS/MS) [25]. This suggests a correlation between low PA levels and malignant gastric cancer progression, i.e., reduced PA levels may promote cancer growth and metastasis. In this study, we investigated the effects of PA on proliferation, apoptosis, invasion, and other malignant biological behaviors in multiple human gastric cancer cell lines, clinical gastric cancer tissue samples, and paracancerous tissue samples, as well as a mouse xenograft model. We believe that our study would help identify potential therapeutic targets for gastric cancer.

## 2. Results

### 2.1. PA Inhibits the Proliferation and Anchorage-Independent Growth of Gastric Cancer Cells

We previously performed a UPLC–MS/MS-based lipidomic analysis of human gastric cancer and paracancerous tissues [25]. The levels of several fatty acids and their derivatives differed significantly between the gastric cancer and paracancerous tissues (*p* < 0.05). Additionally, the PA levels in adjacent tissues were higher than those in the gastric cancer tissues [25].

**Figure 1:**
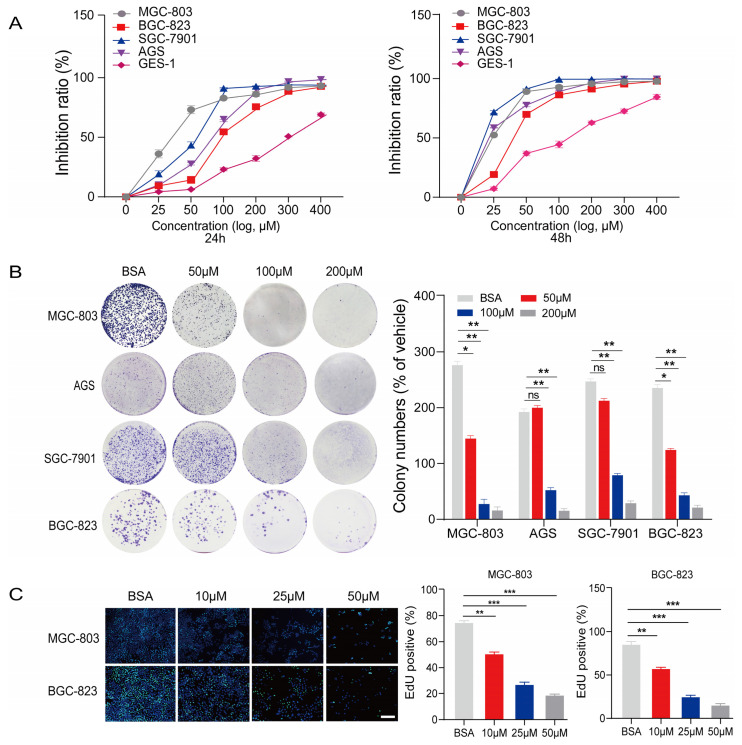
PA inhibits the proliferation and anchorage-independent growth of gastric cancer cells. (**A**) The inhibitory effects of PA on proliferation in human gastric cancer cells and normal gastric epithelial cells were detected using MTT assays after treatment for 24–48 h. Inhibition rates (%) = (A_NC_ − A_PA_)/A_NC_ × 100 A_NC_: absorbance of reaction with without PA, and A_PA_: absorbance of reaction with various concentrations of PA. (**B**) Effects of PA on colony formation in human gastric cancer cells treated for 14 days. (**C**) EdU-DNA assay was performed to detect the effects of PA on MGC-803 and BGC-823 cell proliferation after 18 h (Mean ± SEM, ns nonsignificant, *** *p* < 0.001, ** *p* < 0.01, * *p* < 0.05).

The human gastric cancer cell lines MGC-803, SGC-7901, AGS, and BGC-823, and the normal human gastric epithelial cell line GES-1 were used to observe the effects of PA on cell proliferation. The MTT assay results indicated that 24–48 h treatment with PA inhibited the proliferation of human gastric cancer cell lines in a concentration- and time-dependent manner (Figure 1A). Following the treatment with 150 μM of PA for 24–48 h, MGC-803 cells were inhibited by up to 70.0% and 85.3% at 24 and 48 h, respectively (Figure 1A). These results suggest that PA exerts an anti-proliferative effect on human gastric cancer cells in a time-dependent manner.

We performed a colony formation assay to further monitor the effects of PA on the proliferation of gastric cancer cells. PA inhibited colony formation in MGC-803, SGC-7901, AGS, and BGC-823 cells in a concentration-dependent manner. Notably, the inhibition rates of MGC-803, SGC-7901, AGS, and BGC-823 cells reached 89.0%, 85.7%, 75.0%, and 85.8%, respectively, after treatment with 200 μM of PA for 14 days (Figure 1B). These results were consistent with those of the MTT assay.

We also conducted an EdU-DNA incorporation assay to evaluate the effect of PA (10 μM, 25 μM, and 50 μM) on the proliferation of the MGC-803 and BGC-823 cell lines (Figure 1F). Our results suggested that the number of EdU-positive human gastric cancer cells gradually decreased as the PA concentration increased. After treatment with 50 μM of PA for 18 h, EdU-positive MGC-803 and BGC-823 cells decreased from 80.0% in the BSA control group to 30.6% and 18.9%, respectively, in the PA treatment group (Figure 1C).

### 2.2. PA Inhibits the Invasion of Human Gastric Cancer Cells In Vitro

**Figure 2:**
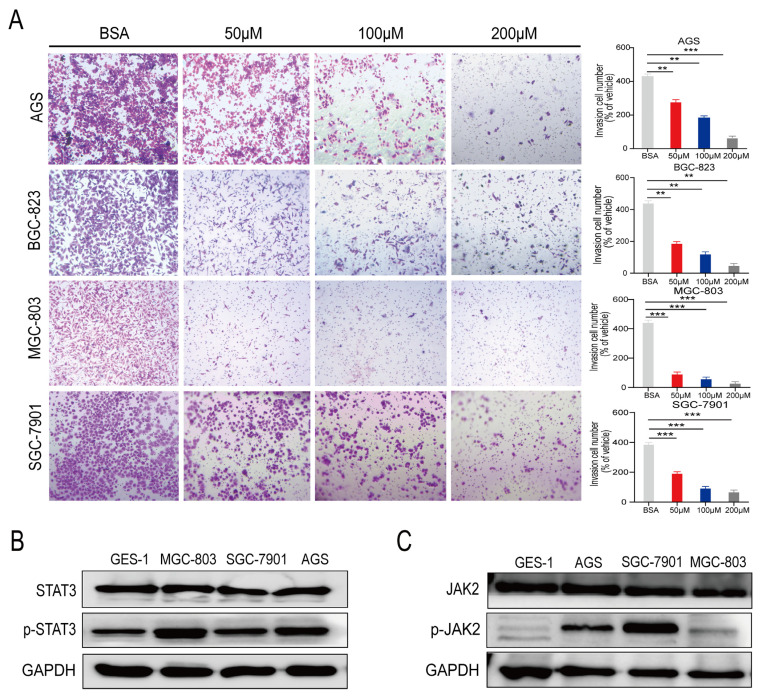
PA inhibits the invasion of human gastric cancer cells in vitro. (**A**) Transwell assays were used to detect the effect of PA on the invasion of the human gastric cancer cells MGC-803, SGC-7901, AGS, and BGC-823 (×100) (Mean ± SEM, *** *p* < 0.001, ** *p* < 0.01). The expression levels of total STAT3 and p-STAT3 (**B**) and total JAK2 and p-JAK2 (**C**) were detected in the human gastric cancer cells MGC-803, SGC-7901, and AGS using Western blotting. (Mean ± SEM, *** *p* < 0.001, ** *p* < 0.01).

We conducted a Transwell assay to investigate the effects of PA on gastric cancer cell invasion. The BSA-treated cells were used as a control. PA inhibited the invasion of MGC-803, SGC-7901, AGS, and BGC-823 cells in a concentration-dependent manner (Figure 2A). The invasive abilities of the four gastric cancer cell lines treated with 200 μM of PA were substantially lower than those of the control group (i.e., 89.8%, 82.5%, 91.7%, and 90.0%, respectively; Figure 2A). In summary, PA blocked proliferation as well as inhibited the invasion of the gastric cancer cells.

Next, we analyzed the levels of various proteins (total and phosphorylated) in the human gastric cells and clinical gastric tissue samples using Western blotting (WB) [25]. The level of total STAT3 protein did not differ among the MGC-803, SGC-7901, AGS, and GES-1 cells; however, the p-STAT3 levels were higher in the MGC-803, SGC-7901, and AGS cells than in the normal gastric epithelial cell line GES-1 (Figure 2B). JAK2 protein levels did not differ among the MGC-803, SGC-7901, AGS, and GES-1 cells. However, the levels of p-JAK2, a key molecule upstream of p-STAT3, were higher in the gastric cancer cell lines than in the normal gastric epithelial cell line GES-1 (Figure 2C).

### 2.3. p-STAT3 Is Highly Expressed in Gastric Cancer Tissues and Cell Lines

**Figure 3:**
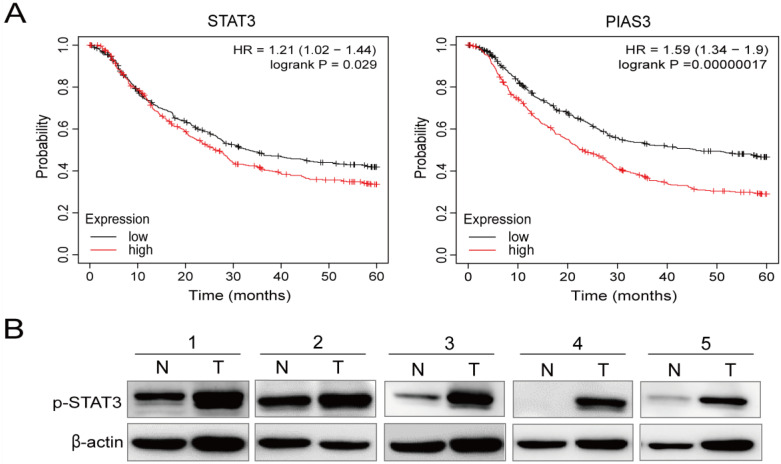

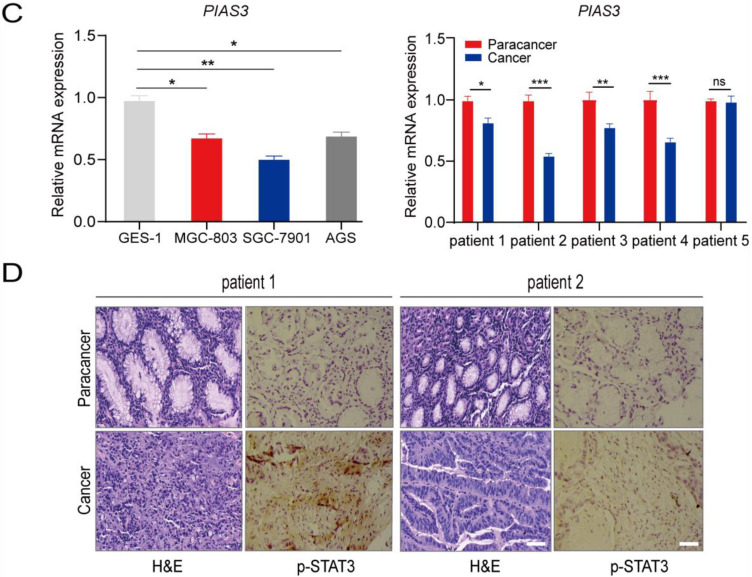
p-STAT3 is highly expressed in human gastric cancer tissues and cell lines. (**A**) Kaplan-Meier curves of overall survival (OS) for total patients reveal relationships between *STAT3* and *PIAS3* expression and clinical gastric cancer. (**B**) The expression of p-STAT3 in the human gastric cancer tissues (T) is higher than that of the matched adjacent tissues (N). (**C**) *PIAS3* mRNA levels are low in the human gastric cancer cell lines and the human gastric cancer tissues. (**D**) H&E staining and the detection of p-STAT3 in the human gastric cancer and paracancerous samples using immunohistochemistry (Mean ± SEM, ns nonsignificant, *** *p* < 0.001, ** *p* < 0.01, * *p* < 0.05, ×100).

We next focused on the relationship between STAT3 and PIAS, as well as the five-year survival rate of patients with gastric cancer. Using the Kaplan–Meier Plotter databases, we observed that low *STAT3* mRNA levels were positively correlated with five-year overall survival (OS; *p* = 0.029). In addition, low *PIAS3* mRNA levels were positively correlated with five-year OS (*p* = 0.00000017) (Figure 3A).

Subsequently, we analyzed the levels of p-STAT3 in the human clinical gastric tissue samples. The WB analyses demonstrated that p-STAT3 levels were higher in the five clinical gastric cancer tissues (T) than in the matched adjacent tissues (N) (Figure 3B). The qRT-PCR analyses indicated that PIAS3 was expressed at lower levels in the human gastric cancer cell lines (MGC-803, SGC-7901, and AGS) and the clinical gastric cancer tissues compared to that in the GES-1 cells and the paracancerous tissues (Figure 3C). Hematoxylin and eosin (H&E) staining helped distinguish the gastric cancer tissues from the paracancerous tissues, and an immunohistochemical analysis revealed that p-STAT3 levels were 60% higher in the gastric cancer samples than in the paracancerous tissues (Figure 3D).

### 2.4. PA Regulates the Expression of p-STAT3 and EMT-Related Proteins

**Figure 4:**
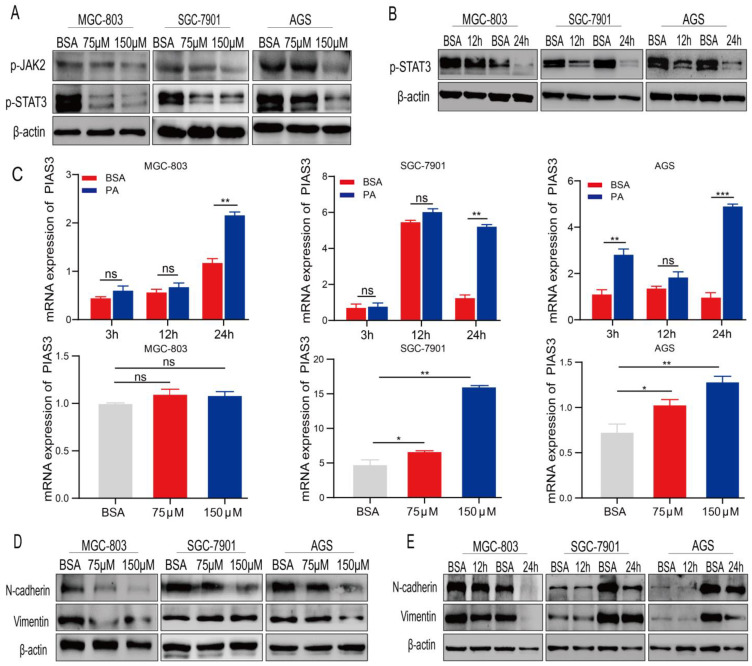
PA-regulated proteins and mRNA expression of p-STAT3 and EMT-related markers. (**A**) The expression of p-STAT3 and p-JAK2 in gastric cancer cell lines was detected using WB after PA treatment. (**B**) The expression of p-STAT3 protein was detected using WB after PA treatment for 12–24 h. (**C**) The qRT-PCR method was used to detect the effect of PA treatment for 12–24 h using different concentrations on the expression of *PIAS3* mRNA in the human gastric cancer cell lines MGC-803, SGC-7901, and AGS. The expressions of N-cadherin and vimentin were analyzed after being treated with different concentrations (**D**) or being treated with 150 μM of PA for 12–24 h (**E**) (Mean ± SEM, ns nonsignificant, *** *p* < 0.001, ** *p* < 0.01, * *p* < 0.05).

We used WB and qRT-PCR analyses to evaluate whether the p-STAT3-PIAS3 signaling axis mediates the anti-gastric cancer effects of PA. The WB results suggested that p-STAT3 levels were lower in the PA-treated human gastric cancer cell lines (MGC-803, AGS, and SGC-7901) than in the BSA-treated cell lines and were significantly correlated with PA concentration. The expression pattern of p-JAK2, which functions upstream of p-STAT3, were lower in the 150μM PA-treated human gastric cancer cell lines (MGC-803, AGS, and SGC-7901) than in the BSA-treated cell lines (Figure 4A). Furthermore, WB analyses demonstrated that p-STAT3 levels decreased in the SGC-7901 and AGS cells after 12–24 h of PA treatment (Figure 4B). As p-STAT3 is a molecular marker of proliferation, our findings suggest that PA blocks the proliferation of human gastric cancer cells by inhibiting p-STAT3 and p-JAK2 expression.

PIAS3 is an inhibitor of p-STAT3. The qRT-PCR analysis revealed that *PIAS3* was higher than in the human gastric cancer cells than in the BSA control group after treatment with 150 μM of PA for 12–24 h or treatment with 75–150 μM of PA for 24 h. However, the mRNA expression level of *PIAS3* in the MGC-803 cells did not change significantly. (Figure 4C). Our results indicated that *PIAS3* mRNA levels increased gradually as the PA treatment duration or the PA concentration increased, consistent with our findings relating to p-STAT3 protein expression in human gastric cancer and normal cells.

Abnormal STAT3 activation can play a critical role in cancer development by influencing the epithelial–mesenchymal transition (EMT) [26]. Accordingly, we evaluated the levels of EMT-related proteins in the gastric cancer cells after PA treatment. The WB analyses indicated that the levels of N-cadherin and vimentin were lower in the PA-treated human gastric cancer cells than in the BSA-treated cells, and the decreases occurred after treatment with 75–150 μM of PA for 24 h (Figure 4D) or 150 μM of PA for 12–24 h (Figure 4E).

### 2.5. PA Induces Apoptosis and Inhibits p-STAT3 Expression in Human Gastric Cancer Cells

**Figure 5:**
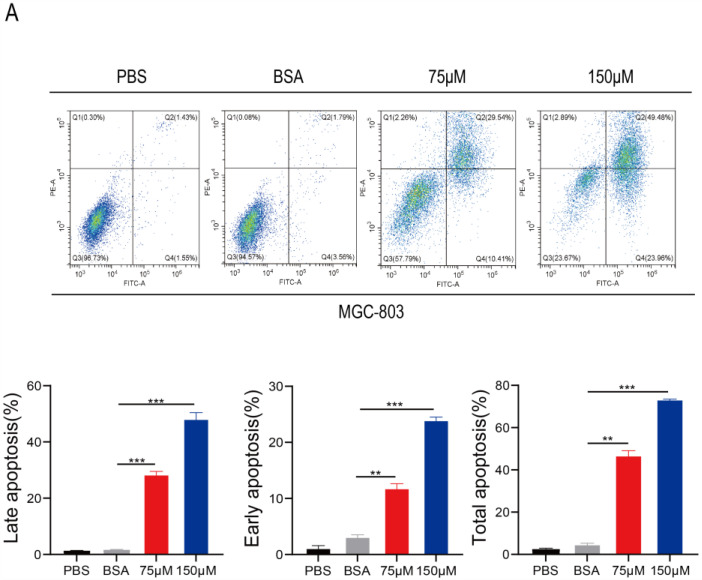

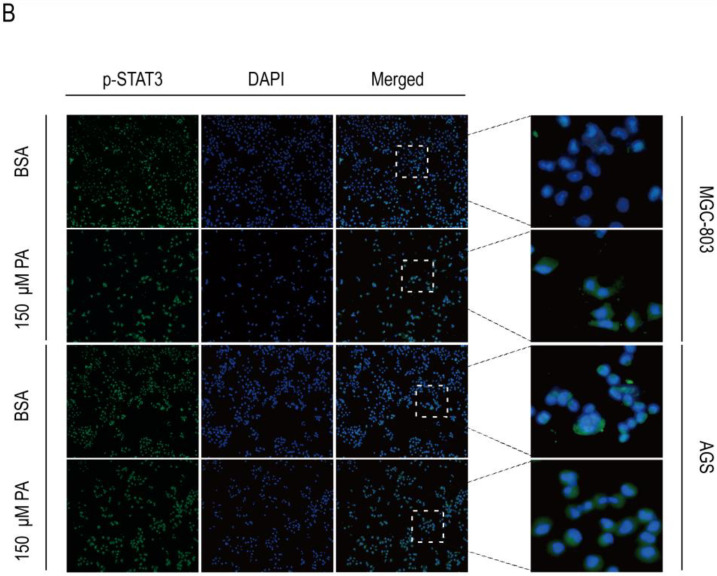
PA induces apoptosis and inhibits p-STAT3 protein expression in human gastric cancer cells. (**A**) Annexin V-FITC/PI apoptosis double staining detection was performed to observe PA-induced human gastric cancer MGC-803 cell apoptosis. Q4 is early apoptosis, Q2 is late apoptosis, and the total apoptosis is Q2+Q4 (**B**) The effects of PA on the nuclear translocation of p-STAT3 protein were detected using immunofluorescence after treatment with PA for 18 h in the human gastric cancer cell lines MGC-803 and AGS (×100) (Mean ± SEM). *** *p* < 0.001, ** *p* < 0.01.

Annexin V-FITC/PI double staining was performed to observe whether PA induces gastric cancer cell apoptosis. The flow cytometry analysis suggested that PA effectively induces apoptosis in gastric cancer cells. Compared to the corresponding rate in the BSA control group, the early apoptosis rates of MGC-803 cells were 10.4% and 23.9% after treatment with 75 μM and 150 μM of PA for 18 h, respectively (Figure 5A), suggesting that PA can induce apoptosis in a concentration-dependent manner [27,28]. Furthermore, we performed a nuclear translocation assay to study the effects of PA on the nuclear expression of p-STAT3. Under normal circumstances, p-STAT3 is located in the nucleus in an activated state and is highly expressed in gastric cancer cells [29]. The immunofluorescence analyses revealed that 150 μM of PA substantially inhibited the expression and translocation of p-STAT3 in the nuclei of MGC-803 and AGS cells compared to that in the control group; p-STAT3 was partially localized in the cytoplasm, and its expression was reduced in the nucleus (Figure 5B). Therefore, PA alters STAT3 protein levels in human gastric cancer cells.

### 2.6. In Vivo Anticancer Activity of PA

**Figure 6:**
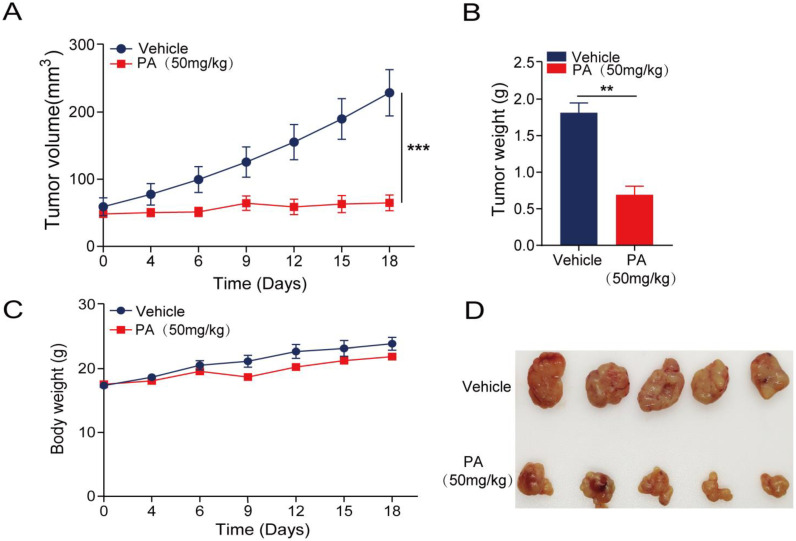
PA shows a strong anticancer effect in vivo. Gastric cancer SGC-7901 cells were injected subcutaneously into male nude mice. When the cancer volume reached ~50 mm^3^, the mice were treated i.p. with 50 μM/kg of PA or BSA (as a control). (**A**) Cancer volumes were measured every other day for 18 days starting from the initiation of treatment. (**B**) The average body weights in each group were measured every other day. (**C**) Growth inhibition rates were measured by weighing the tumors of the experimental animals killed at the end of the experiment after PA was administered to the SGC-7901-bearing mouse xenograft models. (**D**) Representative image of isolated tumors derived from the mice xenograft models after being treated with PA (50 μM/kg/day) or vehicle. (Mean ± SEM, n = 5; *** *p* < 0.001, ** p < 0.01).

To monitor the anticancer activity of PA in vivo, SGC-7901 cells were used to establish a xenograft model in nude mice. Cancer growth was 54.3% lower after treatment with 50 mg/kg of PA than in the control group, suggesting that PA effectively inhibits cancer growth (Figure 6A,B). Furthermore, no significant differences are observed in the body weight curve (Figure 6C), indicating that PA has a low toxicity. The treatment with 50 mg/kg of PA significantly inhibited the tumorigenicity of SGC-7901 cells in nude mice (Figure 6D).

## 3. Discussion

Dysregulated lipid metabolism can cause several metabolic diseases [30], and abnormalities in critical enzymes related to the regulation of fatty acid metabolism and the malignant transformation of cell types are closely related to tumor development [31,32].

PA is a C16 saturated fatty acid that plays distinct roles in different tumors. For example, PA promotes metastasis in some cancers (such as oral squamous cell carcinoma) [14] but inhibits tumor occurrence, development, and malignant metastasis in other cancer types. PA and ceramide can inhibit macrophage infiltration in colorectal cancer via the induction of the EMT [33], whereas C16-D siRNAs inhibit the growth of HT-29/Luc human colorectal cancer cells when subcutaneously injected into nude mice [34]. The findings of the current study demonstrate that PA blocks cell proliferation and invasion in human gastric cancer.

A high ratio (16:1N-7/16:0) of monounsaturated to saturated fatty acids results in increased regulation of fatty acid synthesis and the metabolic activity of lipase [35]. We previously reported that *SCD1* is upregulated in human gastric cancer samples, which may be related to the downregulation of PA in human gastric cancer samples [25].

Although the expression of p-STAT3 in patients with gastric cancer has been systematically reviewed [36,37], the molecular mechanisms underlying STAT3 activation in gastric cancer have not been fully characterized [38,39]. Furthermore, as a critical transcriptional regulator involved in cell proliferation and metastasis, STAT3 plays a vital role in tumorigenesis and tumor development [16]. Our observation that PA inhibits human gastric cancer cell proliferation and metastasis has prompted us to further analyze its effects on STAT3 phosphorylation. In the PA-treated MGC-803 cells, p-STAT3 levels decreased, and its nuclear translocation was substantially inhibited compared to that in the BSA control (Figure 4 and Figure 5). This observation suggests that PA may affect p-STAT3 expression in human gastric cancer cells without affecting total STAT3 and JAK2 levels (Figure 2B,C). Furthermore, these results are consistent with the findings from the in vivo xenograft models in nude mice (Figure 6).

The Kaplan–Meier survival analyses indicated that low *PIAS3* expression was common in patients with gastric cancer, especially those with stage III gastric cancer. The five-year OS was positively correlated with *PIAS3* expression, suggesting that high *PIAS3* expression predicts a better prognosis (Figure 3). *PIAS3* levels were higher in the MGC-803 gastric cancer cells exposed to 150 μM of PA for 12 h than in the cells exposed to BSA, indicating that PIAS3 plays a crucial role in PA-induced inhibition of proliferation and metastasis in human gastric cancer cells (Figure 4). These data are consistent with previous results suggesting that PIAS3 inhibits p-STAT3 biosynthesis [20].

In summary, despite the reported role of STAT3 in numerous cancers, its anti-gastric cancer effects and the underlying molecular mechanisms involving PA have not been established. Our findings demonstrate that PA exerts anti-gastric cancer effects by regulating key molecules (p-STAT3 and its inhibitor PIAS3) in the STAT3-PIAS3 signaling pathway. Furthermore, our preliminary results suggest a strong correlation between the anti-gastric cancer activity of PA and the STAT3-PIAS3 signaling pathway in vitro and in vivo, providing a basis for further research. However, our study has a limitation: we studied PA treatment in isolation (i.e., not combined with other drugs or therapies). Nonetheless, our results provide a preclinical and theoretical basis for further studies on the prevention and prognosis of gastric cancer and the use of PA as a supplement in the clinical treatment of gastric cancer.

## 4. Materials and Methods

### 4.1. Collection of Clinical Tissue Samples

Gastric cancer and paracancerous tissues were collected from patients at the affiliated hospital of Anhui Medical University (Hefei, China). After clinical and histopathological examination, all tissues were diagnosed, and the location, degree of differentiation, depth of infiltration, and lymph node metastasis were determined. The specimens were cataloged, frozen in liquid nitrogen, and stored at −80 °C until further use. None of the patients received chemotherapy or radiotherapy. This study was conducted after obtaining informed consent from the patients and approval from the Ethics Committee of Anhui Medical University (Hefei, China).

### 4.2. Cell Lines and Cell Culture

Human gastric adenocarcinoma cell lines (MGC-803, BGC-823, and SGC-7901) and human normal gastric mucosal epithelial cells (GES-1) were obtained from the Cell Bank of the Typical Culture Preservation Committee of the Chinese Academy of Sciences (Shanghai, China). AGS cells were purchased from the American Type Culture Collection (Manassas, VA, USA). The MGC-803 and SGC-7901 cells were cultured in DMEM (Procell, Wuhai, China). The AGS, BGC-823, and GES-1 cells were cultured in RPMI 1640 (Procell, Wuhai, China) supplemented with 10% FBS (Procell, Wuhai, China) and 0.1% penicillin and streptomycin (Hyclone, Logan, UT, USA). All cells were cultured at 37 °C with 5% CO_2_.

### 4.3. Drug Preparation

Sodium palmitate acid (PA; Across Organics, Thermo Fisher Scientific, Waltham, MA, USA) was stored in distilled water. PA and BSA (Solarbio, Beijing, China) were completely dissolved in a 70 °C water bath to prepare the stock solution (PA:BSA = 6:1). Finally, the PA stock solution was added to a serum-free, BSA-containing medium at various concentrations to obtain the working solution.

### 4.4. Cell Proliferation and Colony Formation Assay

The effects of PA on cell proliferation were examined using an MTT assay (Keygentech, Nanjing, China). Briefly, 4 × 10^3^ or 6 × 10^3^ cells were cultured in 96-well plates overnight and treated with various concentrations of PA for 24–48 h. Subsequently, 200 μL of 20% MTT was added to each well, and the treated cells were incubated at 37 °C for 2–4 h. OD_490_ was measured using a microplate spectrophotometer (Thermo Fisher Scientific, Waltham, MA, USA).

For the colony formation assay, 500 cells were inoculated in six-well plates for 24 h, followed by treatment with BSA (as a control) or PA for 14 days. The cells were fixed with 4% PFA and stained with a 0.1% crystal violet staining fixative (Sangon Biotech, Shanghai, China). Images were obtained for statistical analyses.

### 4.5. EdU-DNA Assay

Cell proliferation was measured using a kFluor488-EdU Kit (Keygen Biotech, Nanjing, China) [40]. The cells (6 × 10^3^) were cultured in 96-well plates for 24 h. Following treatment with PA, an EdU-labeling reagent was added to the cells. The nuclei were stained with 4′,6-diamidino-2-phenylindole (DAPI)(Beyotime, Shanghai, China). A fluorescence microscope (Observer A1; Zeiss, Oberkochen, Germany) was used to observe EdU-positive cells, and Image J (NIH, Bethesda, MD, USA) was used for fluorescence quantification.

### 4.6. Transwell Cell Invasion Assay

Cell invasion was evaluated in an 8 μm Transwell chamber. Briefly, Matrigel was placed at the bottom of the chamber for 30 min at 37 °C. The cells (8 × 10^4^) were seeded into the insert chamber for 24 h. After PA and BSA (as control) treatment for 24 h, cells passing through the bottom of the membrane were stained with 0.1% crystal violet in 10% formalin and observed under a microscope (Olympus, Tokyo, Japan).

### 4.7. WB Analysis

WB was performed following standard procedures [25,41]. The gastric cancer tissues were homogenized using a grinding-tissue homogenizer. A RIPA lysis buffer (Solarbio, Beijing, China) was used to extract proteins from the cell lines or human tissues. Protein concentration was measured using the BCA method (Beyotime, Shanghai, China). Equal amounts of proteins were separated using 10–12% SDS-PAGE and transferred to PVDF membranes (Millipore, Billerica, MA, USA). The membranes were incubated with 5% skim milk, followed by incubation with primary antibodies (antibodies against STAT3, (124H6) Mouse mAb, cat# 9139T, Cell Signaling Technology, Danvers, MA, USA; Phospho-STAT3, (Tyr705) (D3A7) XP^®^ Rabbit mAb, cat#9145S, Cell Signaling Technology, Danvers, MA, USA; JAK2, cat#AF6022, Affinity; Phospho-JAK2, (Tyr1007), cat#AF3022, Affinity; N-cadherin, cat#E-AB-70061, Elabscience, Wuhan, China; Vimentin, cat#E-AB-70081, Elabscience) overnight at 4 °C. The samples were then incubated with a HRP-labeled secondary antibody (Elabscience, Wuhan, China) for 1 h at 25 °C. Finally, protein levels were detected using ECL reagents (GE Healthcare, Shanghai, China). Anti-GAPDH and anti-β-Actin antibodies (Elabscience, Wuhan, China) were used as internal controls. Original images of WB figures please see Supplementary File.

### 4.8. RNA Isolation and qRT-PCR

Total RNA was extracted using a TRIzol reagent (Kangwei Shiji, Beijing, China) following a previously described procedure [39,40]. cDNA was synthesized using the RevertAid First Strand cDNA Synthesis kit (Thermo Scientific, Waltham, MA, USA), followed by amplification by target-specific primers and PCR Master Mix (TaKaRa, Beijing, China). The cycling conditions were as follows (40 cycles): pre-denaturation at 95 °C for 3 min, denaturation at 95 °C for 30 s, and annealing at 60 °C for 30 s. *GAPDH* mRNA levels were used as an internal standard control. The primer sequences were as follows: *PIAS3* (Forward: TGTCACCATGAAACCATTGC; Reverse: AGGTAAAGTGCGCTTCCTCA) and *GAPDH* (Forward: GACCTCTATGCCAACACAGTGC; Reverse: GTACTCCTGCTTGCTGATCCAC).

### 4.9. Flow Cytometric Analysis of Cell Apoptosis

Rates of apoptosis in the human gastric cancer cells were evaluated using the Annexin V-FITC/PI Apoptosis Detection kit (Beyotime, Shanghai, China) [40,41]. Briefly, 1 × 10^5^ MGC-803 cells were cultured in 6-well plates overnight. Subsequently, the cells were treated with 75–150 μM of PA for 18 h and collected with 0.1% sodium citrate containing 0.1% Triton X-100. The cells were then treated with Annexin V-FITC and incubated in the dark for 15–25 min at 37 °C. Apoptotic cells were detected using flow cytometry (Beckman-Coulter, Brea, CA, USA).

### 4.10. Immunofluorescence Analysis

Nuclear p-STAT3 protein levels in the gastric cancer cells were measured using immunofluorescence [40,42]. Briefly, 1 × 10^5^ MGC-803 cells were cultured in 6-well plates overnight, followed by treatment with 150 µM of PA for 18 h, fixation with 4% paraformaldehyde for 30 min, and sealing with a 10% goat serum containing 1% BSA and 0.1% Triton X-100 for 1 h at 25 °C. The cells were incubated with an anti-p-STAT3 antibody for 24 h at 4 °C, followed by incubation with a fluorescein isothiocyanate-conjugated secondary antibody, and DAPI was used to stain the nuclei for 1 h. Changes in p-STAT3 nuclear translocation were detected under a fluorescence microscope (Olympus, Tokyo, Japan).

### 4.11. In Vivo Human Cancer Xenograft Models

BALB/c nude mice (male, 6–8 weeks, 18–22 g) were purchased from Jiangsu Jicui Yaokang Biological Co. Ltd. (Nanjing, China). All mice were housed with free access to food and water under controlled light conditions (21 °C, 55% humidity, and 12 h light/dark cycle). The mice were first anesthetized with fluorine chlorine hydride and then sacrificed by exsanguination. All mouse experiments were approved by the Experimental Animal Ethics Committee of AHMU (Hefei, China). All experiments involving mice were performed per the ARRIVE reporting guidelines.

Approximately 5 × 10^6^ SGC-7901 cells were injected into the flanks of the nude mice to establish a human gastric cancer syngeneic s.c. xenograft model. When the cancer volume reached approximately 50 mm^3^, PA was administered via i.p. injections once daily at the designed doses (50 mg/kg or vehicle). Cancer growth and mouse body weights were measured every other day throughout the experiment. Cancer volumes (mm^3^) were calculated as 0.5 × length (mm) × width^2^ (mm). The mice were then sacrificed, and the cancer tissues were collected.

### 4.12. In Vivo H&E Staining and Immunohistochemical Analysis

H&E staining was performed according to a previously described method [42,43]. Briefly, the human gastric cancer and paracancerous tissues were fixed and embedded in paraffin. Subsequently, 4 µM sections were incubated with a H&E solution, and histopathological changes were observed. Next, the sections were incubated with a primary antibody against p-STAT3, followed by the corresponding secondary antibody. The sections were visualized using peroxidase-DAB under an inverted microscope (Olympus, Tokyo, Japan). Quantification was performed using Image J software (NIH, Bethesda, MD, USA).

### 4.13. Kaplan–Meier Plotter Analysis

We evaluated the prognostic value of *STAT3* and *PIAS3* expression for recurrence-free survival in patients with gastric cancer using the Kaplan–Meier Plotter online database. Briefly, the patient samples were divided into high- and low-expression groups using the median value as the threshold. *STAT3* and *PIAS3* were used to obtain five-year survival plots in which the number of patients at risk was measured. The hazard ratios (HR; 95% confidence interval) and log-rank *p*-values were analyzed.

### 4.14. Statistical Analysis

Data are presented as means ± standard error of the mean (SEM) or standard deviation (SD). The differences between the two groups were analyzed using Student’s *t*-tests. For comparisons among more than two groups, one-way or two-way ANOVA was used, followed by Tukey’s post hoc tests. Statistical significance was set at *p* < 0.05.

## 5. Conclusions

In conclusion, we investigated the effects of PA on human gastric cancer cell lines and combined them with the results from clinical gastric cancer and paracancerous tissue samples. Our findings demonstrated that PA exerted anti-gastric cancer effects by regulating key molecules in the signal transduction and activation of STAT3-PIAS3 signaling pathway. Our results serve as a foundation for further research on the correlation between the anti-gastric cancer activity of PA and the STAT3-PIAS3 signaling pathway. Our results also represent a critical step toward understanding gastric cancer prevention and prognosis and promoting PA supplementation as a gastric cancer treatment.

## Data Availability

All data are available from the corresponding authors upon request.

## Supplementary Materials

The following are available online at https://www.mdpi.com/article/10.3390/cancers15020388/s1, The supplementary file: original images of western blot figures.
