# Supplementary figures and images for: Palmitic Acid Inhibits the Growth and Metastasis of Gastric Cancer by Blocking the STAT3 Signaling Pathway
Source: Cancers (Basel). 2023 Jan 6;15(2):388. doi: 10.3390/cancers15020388 (PMC9856364; doi:10.3390/cancers15020388)

Figure 2B

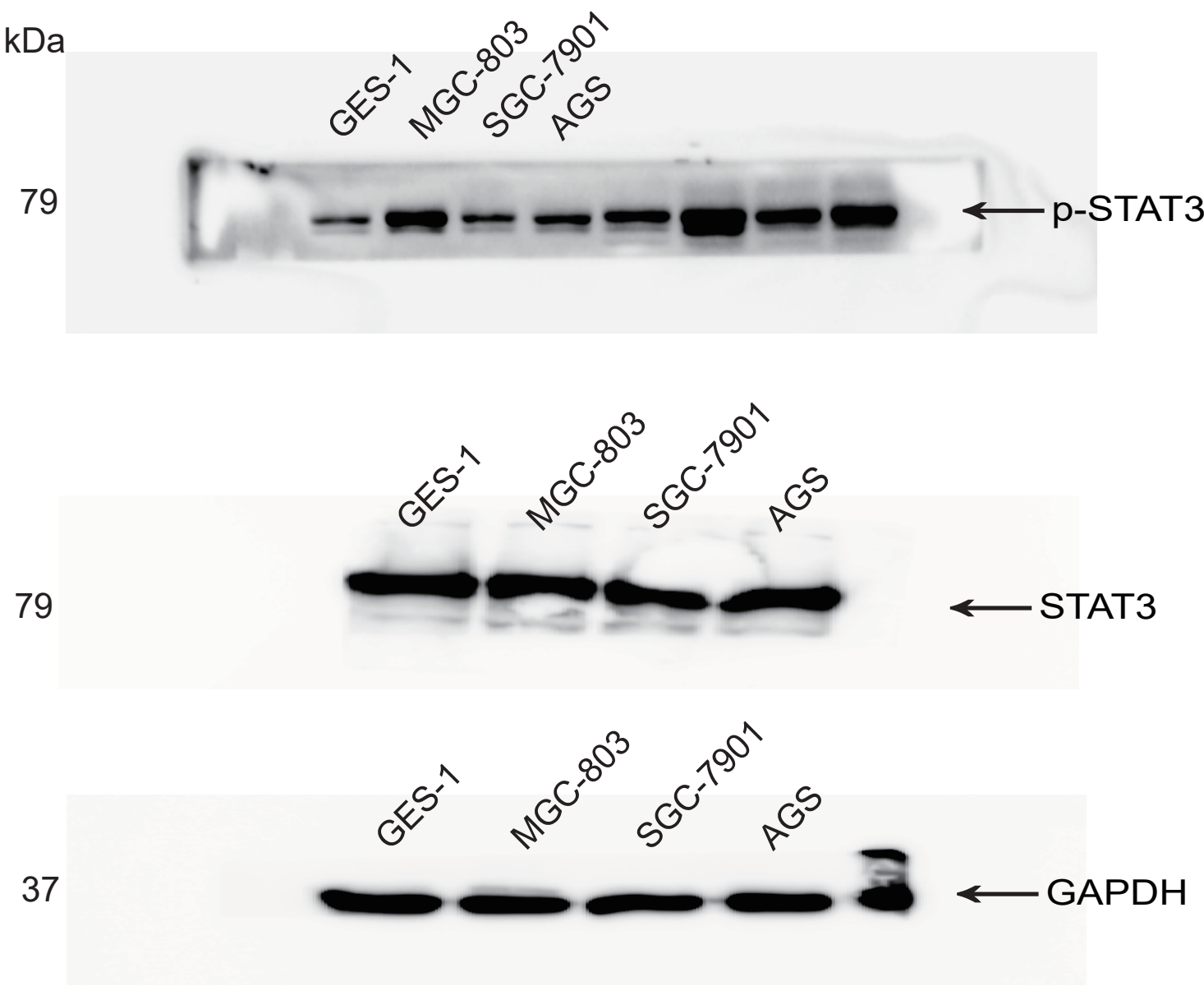

Figure 2C

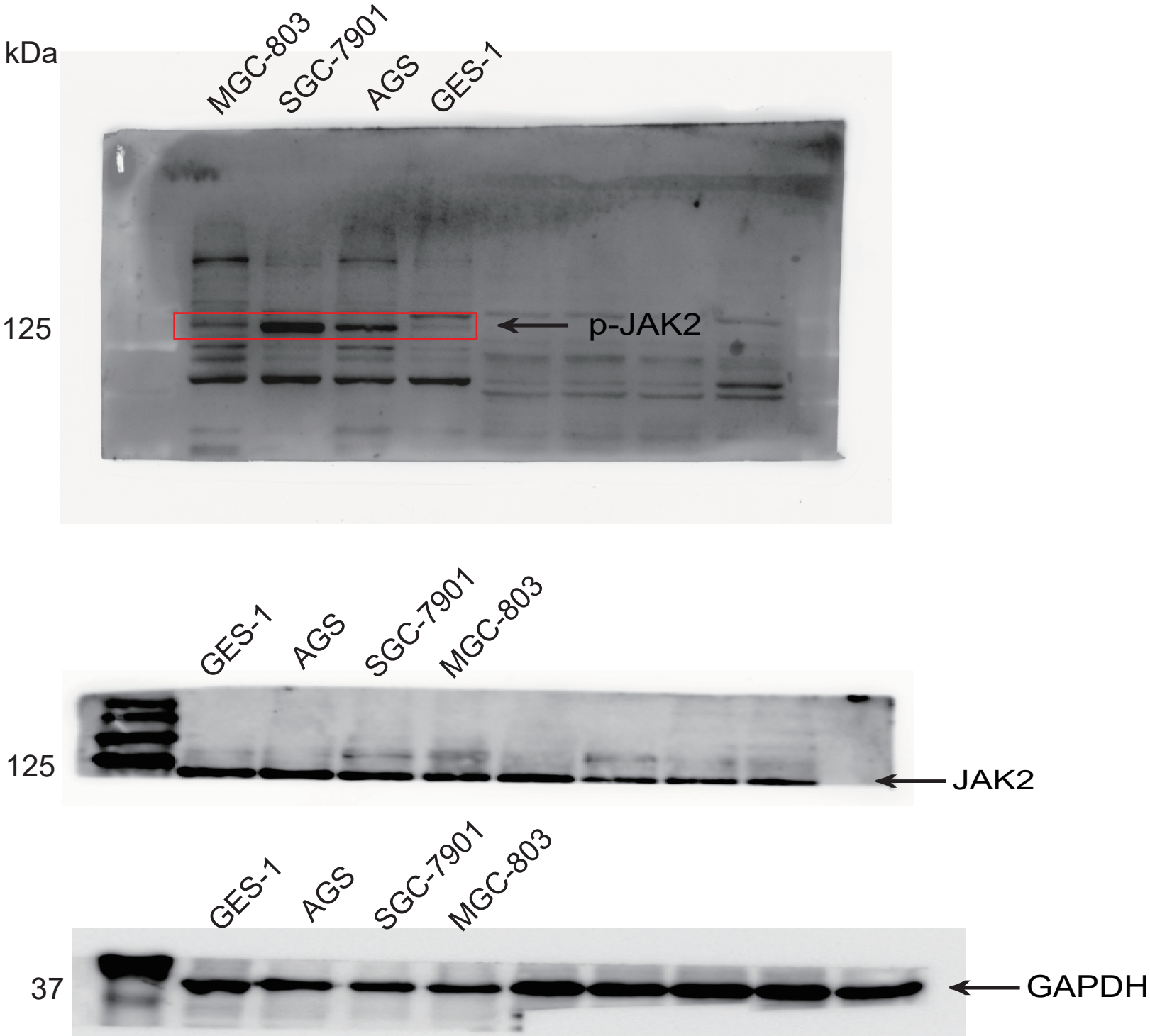

Supplement: Supplementary file 1 [file cancers-15-00388-s001.zip › Fig S2 WB .pdf]

**Figure 3B**

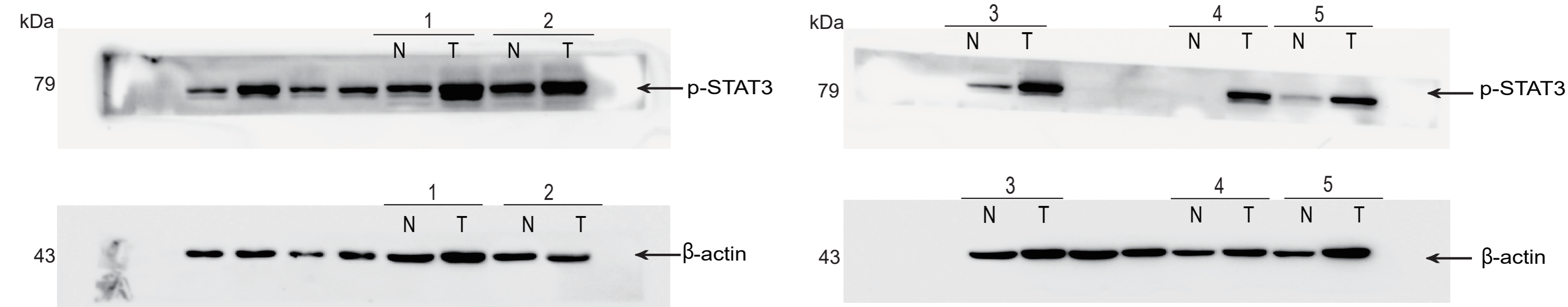

Supplement: Supplementary file 1 [file cancers-15-00388-s001.zip › Fig S3C WB.pdf]

Figure 4A

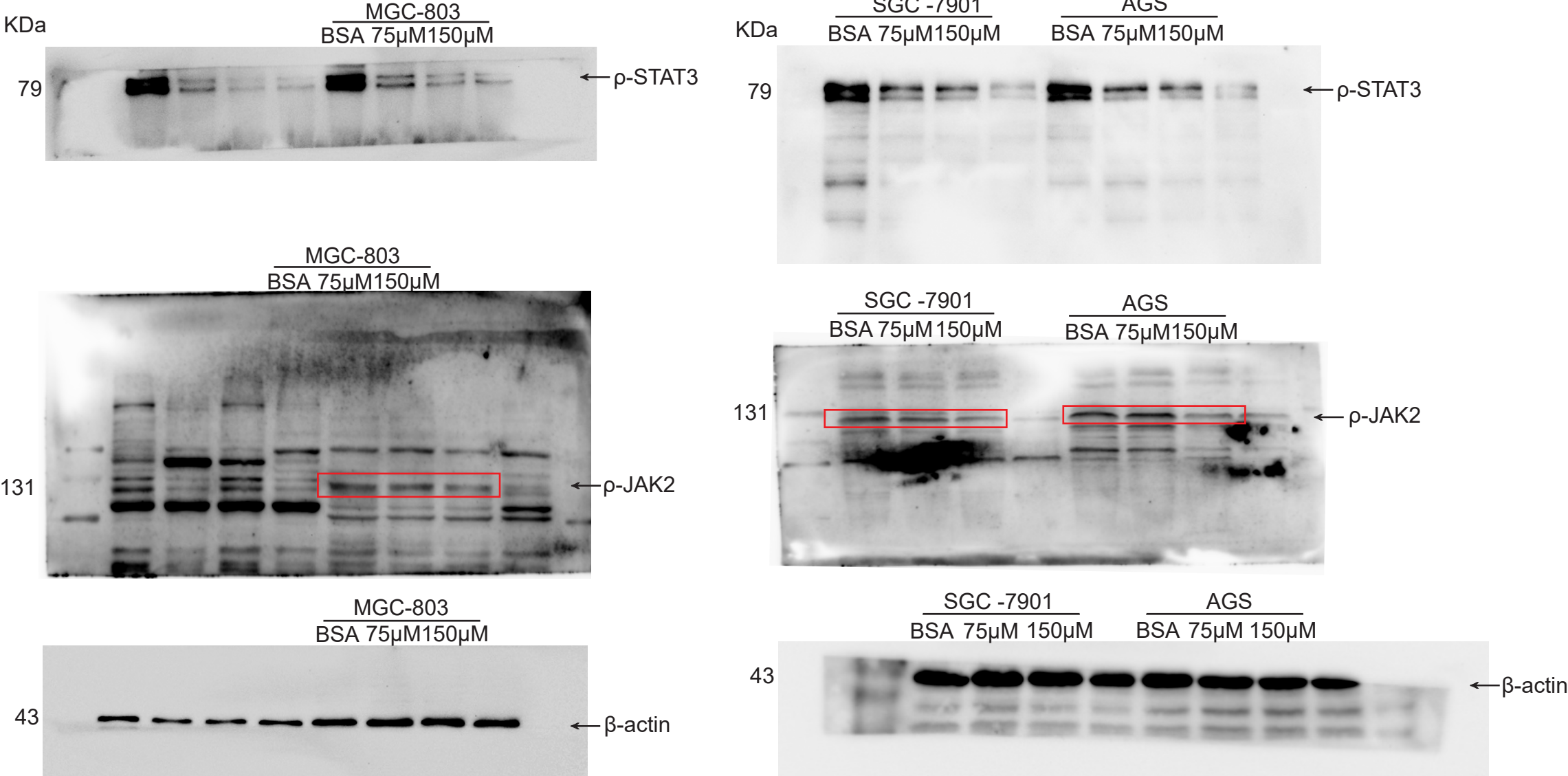

Supplement: Supplementary file 1 [file cancers-15-00388-s001.zip › Fig S4A WB.pdf]

Figure 4B

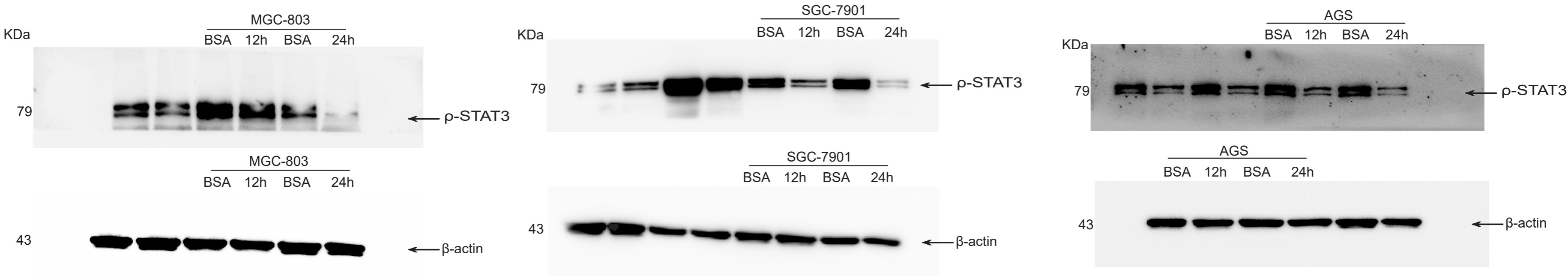

Supplement: Supplementary file 1 [file cancers-15-00388-s001.zip › Fig S4B WB.pdf]

Figure 4D

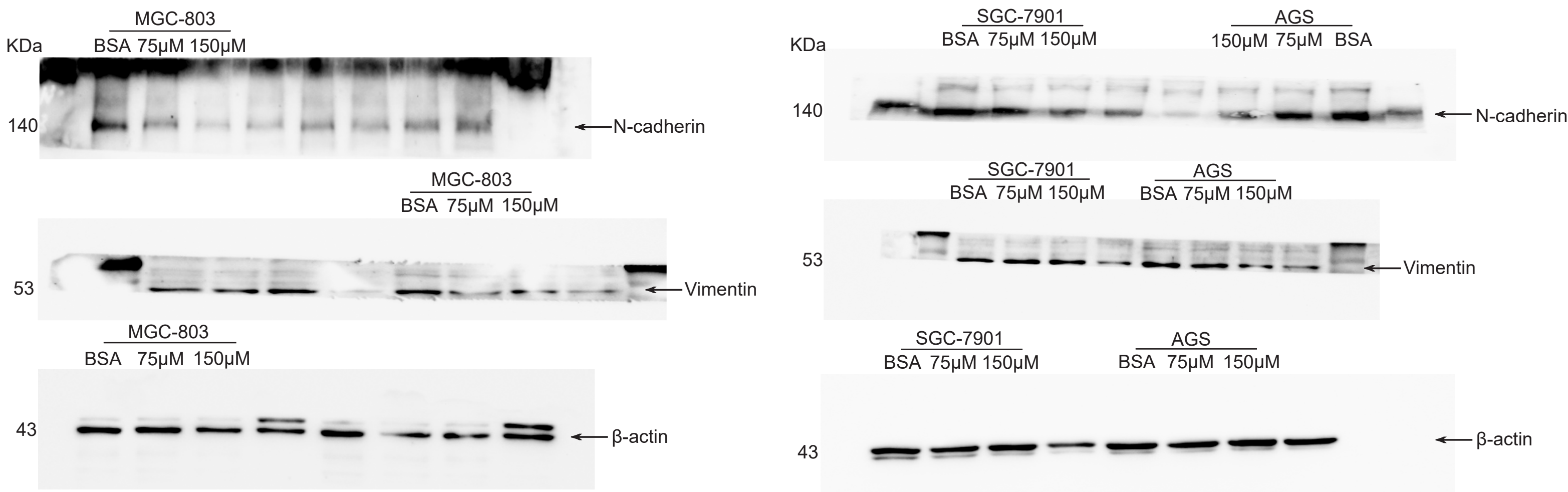

Supplement: Supplementary file 1 [file cancers-15-00388-s001.zip › Fig S4D WB.pdf]

Figure 4E

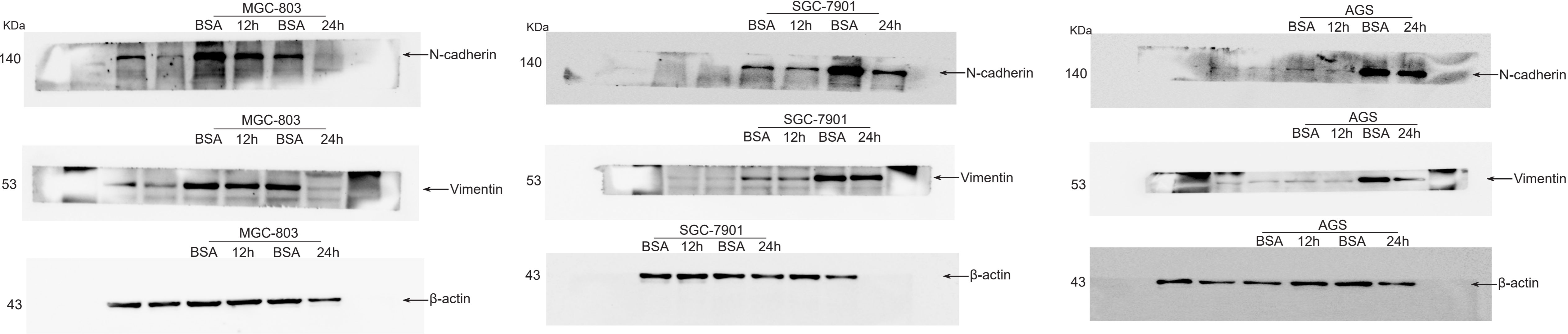

Supplement: Supplementary file 1 [file cancers-15-00388-s001.zip › Fig S4E WB.pdf]
